# Targeted Mutation of Nuclear Bone Morphogenetic Protein 2 Impairs Secondary Immune Response in a Mouse Model

**DOI:** 10.1155/2015/975789

**Published:** 2015-08-23

**Authors:** Daniel S. Olsen, Wesley A. Goar, Brandt A. Nichols, K. Tyson Bailey, S. Loyd Christensen, Kayla R. Merriam, Paul R. Reynolds, Eric Wilson, K. Scott Weber, Laura C. Bridgewater

**Affiliations:** ^1^Department of Microbiology and Molecular Biology, Brigham Young University, Provo, UT 84602, USA; ^2^Department of Physiology and Developmental Biology, Brigham Young University, Provo, UT 84602, USA

## Abstract

We recently identified a nuclear variant of the BMP2 growth factor, called nBMP2. In an effort to understand the function of this variant protein, we generated a mouse line in which BMP2 is expressed and functions normally, but nBMP2 is excluded from the nucleus. This novel mutation allows the study of nBMP2 without compromising BMP2 function. To determine whether nBMP2 plays a role in immune function, we performed a series of experiments in which we compared mouse survival, organ weights, immune cells numbers, and bacterial load in wild type and nBmp2NLS^tm^ mice following primary and secondary challenges with *Staphylococcus aureus*. Following primary challenge with *S. aureus*, wild type and nBmp2NLS^tm^ mice showed no differences in survival or bacterial load and generated similar numbers and types of leukocytes, although mutant spleens were smaller than wild type. Secondary bacterial challenge with *S. aureus*, however, produced differences in survival, with increased mortality seen in nBmp2NLS^tm^ mice. This increased mortality corresponded to higher levels of bacteremia in nBmp2NLS^tm^ mice and to a reduced enlargement of mutant spleens in response to the secondary infection. Together, these results suggest that the recently described nuclear variant of BMP2 is necessary for efficient secondary immune responses.

## 1. Introduction

Our group recently reported that a nuclear variant of the BMP2 growth factor is produced by alternative translation from a downstream start codon [1]. This alternate translation results in a truncated protein that lacks the N-terminal signal peptide and is thus not recognized by the signal recognition particle (SRP) for delivery to the secretory pathway. Instead, the alternative protein is translated in the cytoplasm. Had it entered the secretory pathway, the protein would have been cleaved at a furin-type proprotein convertase recognition sequence to release the mature BMP2 growth factor. In the absence of such cleavage, however, a bipartite nuclear localization signal that overlaps the proprotein convertase recognition sequence directs translocation of the variant protein to the nucleus [1]. Both nuclear and secreted BMP2 can be produced from the same mRNA transcript, and it is therefore possible that some of the functions previously attributed to secreted BMP2 may in fact be performed by nuclear BMP2 (nBMP2).

To distinguish the functions of secreted BMP2 from nBMP2, we generated a mouse strain containing a targeted mutation in which the conventional growth factor BMP2 is secreted and signals normally, but nBMP2 is excluded from the nucleus by means of a three-amino-acid substitution in the nuclear localization signal [2]. The resulting nBmp2NLS^tm^ mice are normal in size, morphology, and fertility, but males exhibit slowed relaxation of skeletal muscles after stimulated contractions. (Females were not tested.) The slowed relaxation phenotype is attributed to a slowed return of Ca^2+^ from the cytosol to the sarcoplasmic reticulum stores at the end of each muscle contraction [2–4].

As in skeletal muscle, intracellular Ca^2+^ transport regulates the activation and differentiation signaling pathways of many immune cells, including T cells, B cells, dendritic cells, and macrophages [5–7]. Based on our findings of disrupted Ca^2+^ transport in skeletal muscle of nBMP2-deficient nBmp2NLS^tm^ mice, we hypothesized that the immune response might also be impaired in nBmp2NLS^tm^ mice [5]. Having female mice available as a result of breeding mice for a different, male-specific study, we decided to explore this immune response hypothesis in female mice by examining the presence and abundance of several immune cell types including neutrophils, monocytes, and several lymphocyte subsets. The overall functionality of the immune response was determined by challenging nBmp2NLS^tm^ and wild type mice with systemic* Staphylococcus aureus* (*S. aureus*) infection and assessing bacteremia and mouse mortality. In this study we identified impaired clearance of bacteria in nBmp2NLS^tm^ mice when compared to wild type mice. Additionally, nBmp2NLS^tm^ mice experienced increased mortality following secondary* S. aureus* infection when compared to wild type controls. These results demonstrate the necessity of the nuclear variant of BMP2 for optimal secondary immune responses.

## 2. Materials and Methods

### 2.1. Research Animals

This study was carried out in strict accordance with the recommendations in the Guide for the Care and Use of Laboratory Animals of the National Institutes of Health. The protocol was approved by the Institutional Animal Care and Use Committee (IACUC) of Brigham Young University (protocols numbers 120105 and 120305).

Heterozygous nBmp2NLS^tm^ mice were bred in the Brigham Young University specific pathogen free (SPF) animal facility. After weaning, mice were genotyped as previously described [2] and transferred to the non-SPF animal care facility where they were housed with 4 or fewer animals per cage and maintained throughout the duration of the study on a standard chow and water diet* ad libitum* in a temperature-controlled (21-22°C) room with a 12 : 12 hour light : dark cycle. All experiments were performed with female mice between the ages of 6–8 months.

### 2.2. Bacterial Infections

*S. aureus*, ATCC strain 12600, was cultured in tryptic soy broth. Organism identification was verified through morphology, Gram stain, catalase test, and mannitol fermentation. Subsequent passages were created by alternating between a standard streak plate and a broth culture. Streak plates were performed on Mannitol Salt Agar (MSA) (Alpha Biosciences), and broth cultures were prepared in LB Broth (BD Biosciences). All cultures were incubated for 24 hours at 37°C and stored at 4°C. Broth cultures were placed on a rotating platform at 120 rpm during incubation. Culture titer was determined by standard plate count.

Mice were infected systemically with* S. aureus* by tail vein injection of approximately 200 *μ*l using a 1 mL syringe and a 27-gauge needle.* S. aureus* culture was diluted to the desired concentration in phosphate buffered saline (PBS) before injection, and control mice were injected with PBS only. Injection dose was verified following injection by standard plate count.

Initial infections were performed at a dose of 3 × 10^5^ CFU/g body weight. This dose was used for all experiments that are shown at 3 days after primary infection. Due to high mortality in both experimental groups at this dose, a lower dose of 1 × 10^4^ CFU/g was used for all subsequent primary infections.

Secondary infection experiments were performed by infecting mice with a priming dose of 1 × 10^4^ CFU/g on day 0 (primary infection), then infecting again with a dose of 3 × 10^5^ CFU/g on day 35 (secondary infection). Analyses of secondary infection effects were performed three days later, on day 38 after primary infection.

### 2.3. Bacterial Load Measurements

Mice were euthanized using CO_2_ inhalation followed by cervical dislocation. Blood samples were taken by cardiac puncture and placed in PBS containing ethylenediaminetetraacetic acid (EDTA) at a final concentration of 1.8 mg/mL to prevent clotting. Mice were then dissected to obtain liver, kidney, spleen, and inguinal lymph nodes. Solid tissue samples were weighed, placed in PBS (5–10 mL for liver, spleen, and kidney samples; 3–5 mL for lymph node samples), and homogenized using a dounce homogenizer.

Tissue samples were serially diluted in tenfold increments (0.5 mL into 4.5 mL) using PBS as needed to obtain colony counts within countable range. 100 *μ*L aliquots were plated in triplicate on MSA to select for growth of* S. aureus*, and plates were incubated for 24–48 hours at 37°C. Bacterial load was calculated as CFU/mL for blood samples and CFU/g for solid tissue samples.

### 2.4. Histology

Spleen samples were fixed overnight in 4% paraformaldehyde in PBS at 4°C. After paraformaldehyde fixation, samples were dehydrated using increasing concentrations of ethanol and processed in a Citadel 1000 tissue processor. Samples were then embedded in paraffin wax and sectioned at 5–7 *μ*m. Sections were stained using hematoxylin and eosin (H&E), visualized on a Zeiss Imager A.1 brightfield microscope, and imaged using a Zeiss AxioCamHRc with AxioVision software, release 4.8.2.

### 2.5. Antibody Measurements

Assays to measure total antibody levels were performed by coating ELISA plates (Nunc) with 2 *μ*g/mL of capture antibody diluted in PBS and coated overnight at 4°C. The following coating antibodies were used: rat anti-mouse IgA (clone C10-3) and rat anti-mouse IgM (clone II/41) (BD Biosciences). For IgG detection, polyclonal goat anti-mouse IgG was used (KPL Gaithersburg, Maryland). Each coating antibody was diluted at 1 : 250 in PBS and incubated overnight at 4°C. Following coating, all plates were washed extensively with PBS and then incubated in blocking buffer for 30 minutes at room temperature. Isotype standards (BD Biosciences) and serum samples were diluted in blocking buffer and then serially diluted and incubated for one hour at room temperature. Plates were then washed extensively and peroxidase labeled goat anti-mouse IgA, anti-mouse IgG, and anti-mouse IgM (all from KPL) diluted at 1 : 1000 were added and incubated at RT for 1 hour. Plates were again washed extensively. Color development was realized through addition of 50 *μ*L of OptEIA TMB substrate (BD Biosciences). OD readings were taken at 450 nm. Antibody concentrations were calculated through the use of a standard curve, and unpaired Student's *t*-test was performed to determine statistical significance.

Antigen-specific immune responses were detected using flow cytometry to measure the binding of* S. aureus*-specific antibodies to intact bacteria. Briefly,* S. aureus* (ATCC strain 12600) was grown overnight on MSA plates at 37°C. Bacteria were then dispersed in PBS containing 10% human serum and incubated for 1 hour on ice. Bacterial samples (100 *μ*L) were then cultured with 3 *μ*L of preimmune or postimmune (day 38) serum for 1 hour on ice. Samples were washed by centrifugation in 5 mL of sterile PBS and incubated with FITC-labeled goat anti-mouse IgG antibody (BioLegend, San Diego, CA). Samples were washed in PBS before flow cytometry analysis. Antigen-specific antibody levels in serum from wild type and mutant mice were determined by subtracting the value of background fluorescence (measured in preimmune serum from uninfected mice) from samples stained with postimmune serum. Levels of antigen-specific anti-*S. aureus* antibody binding were compared between groups using unpaired Student's *t*-test with a value of *p* < 0.05 being considered statistically significant.

### 2.6. Leukocyte Analysis by Flow Cytometry

Mice were dissected to obtain spleen, thymus, and lymph node (inguinal, mesenteric, and lumbar) samples. Tissues were ground between frosted microscope slides and filtered through 70 *μ*m nylon mesh (BD Biosciences) to prepare single cell suspensions in PBS. Cells were then centrifuged and resuspended in 5 mL of red blood cell lysis buffer (388 mM NH_4_Cl, 29.7 mM NaHCO_3_, and 25 *μ*M Na_2_EDTA) for 5 minutes. After red blood cell lysis, samples were centrifuged and resuspended in 500 *μ*L of PBS. To reduce nonspecific binding of antibodies, an FC receptor block was performed by adding anti-mouse CD16/32 antibody to cells (1 *μ*L Ab per 100 *μ*L of cells) and incubating at room temperature for 15 minutes. Samples were then centrifuged and resuspended in antibody staining solution (1 *μ*L antibody in 200 *μ*L PBS) for 30 minutes. All antibodies were from eBiosciences—see Table 1 for antibody identities and groupings. Following staining, samples were rinsed once with PBS and measured on a FACSCanto Flow Cytometer (BD Biosciences). Results were analyzed using Summit Software v4.3 (Beckman Coulter) and FlowJo v10.0.6 (Tree Star). The average size of wild type and mutant spleen cells was compared on the basis of the mean fluorescent intensity of forward scatter gates.

### 2.7. Calcium Flux Analysis

Mice were dissected to obtain spleen, thymus, and lymph node (inguinal, mesenteric, and lumbar) samples. Tissues were ground using frosted microscope slides and filtered through 70 *μ*m nylon mesh (BD Biosciences) to prepare single cell suspensions in PBS. Cells were centrifuged and resuspended in 5 mL of red blood cell lysis buffer (388 mM NH_4_Cl, 29.7 mM NaHCO_3_, and 25 *μ*M Na_2_EDTA) for 5 minutes. After red blood cell lysis, samples were centrifuged and resuspended in 1 mL PBS.

Approximately 5 × 10^6^ cells were resuspended in 500 *μ*L media (RPMI without phenol red +2% FCS). 500 *μ*L of Fluo4 loading solution (2 *μ*M Fluo4, 0.02% pluronic acid in RPMI without phenol red) was added to each cell suspension followed by room temperature incubation in the dark for 30 min. Samples were washed twice with media (500 *μ*L, then 400 *μ*L) and incubated in dark at room temperature for another 15 min. Samples were then stained with antibodies (diluted 1 : 200) specific for CD4, CD8, and B220 (eBioscience; GK1.5, RA3-6B2, and 53-6) on ice for 15 minutes. After staining, cells were washed with 500 *μ*L media and resuspended in 500 *μ*L Ringer solution (150 mM NaCl, 10 mM glucose, 5 mM HEPES, 5 mM KCl, 1 mM MgCl_2_, and 2 mM CaCl_2_) warmed to 37°C.

The stained cells were analyzed on a FACSCanto Flow Cytometer (BD Biosciences). Each sample was measured for 30 seconds to establish baseline calcium flux. 1 *μ*L of ionomycin was then added to the sample and the samples were measured for 30 more seconds. Data was analyzed using FlowJo v10.0.6 (Tree Star) by gating into leukocyte population, CD4+, CD8+, and B220+ cells. To calculate calcium flux, median Fluo 4 levels for each sample were normalized to the baseline level, resulting in a percent increase over baseline for each condition.

### 2.8. Statistical Analysis

Results were analyzed to determine statistical significance using unpaired, two-tailed Student's *t*-test with a significance level of *α* = 0.05. Statistically significant differences are marked in figure panels and labeled with the *p* value of the difference.

## 3. Results

### 3.1. Spleen Size Was Reduced in Naïve nBmp2NLS^tm^ Mice, but Leukocyte Cell Type Distribution in the Spleen Was Normal

**Figure 1:**
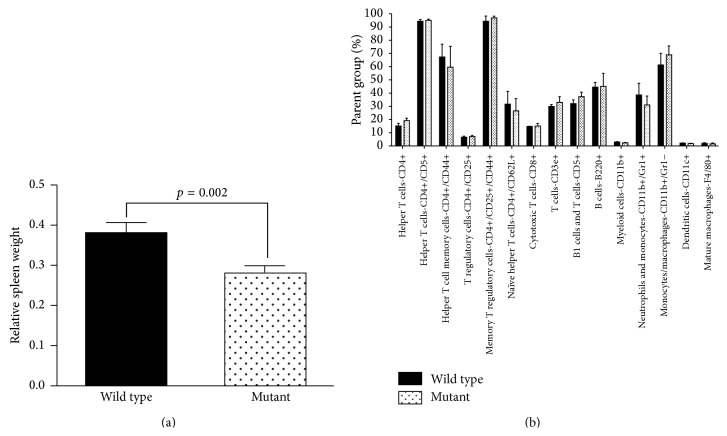
Spleen size is reduced in naïve nBmp2NLS^tm^ compared to wild type mice. (a) Spleen weight as a percentage of total body weight in naïve wild type and mutant adult mice between 6 and 8 months of age. Values shown are average ± SE (*n* = 20 per group). (b) Spleen leukocyte types were measured using flow cytometry. Values are shown as a percentage of the parent group, with the largest parent group being total spleen leukocytes. For example, the columns labeled CD4+ represent the percent of total leukocytes that were CD4+. If subgroups were analyzed, the parent group is given before the slash mark. For example, CD4+/CD5+ represents the percent of CD4+ cells that were also CD5+. CD4+/CD25+/CD44+ represents the percentage of CD4+ and CD25+ cells that are also CD44+ (cells that are positive for both CD4 and CD25 are the parent group). Values shown are average ± SE (*n* = 3 per group).

Liver, kidneys, spleen, and lymph nodes from naïve (uninfected) wild type and nBmp2NLS^tm^ mutant mice were examined and weighed to determine whether differences were detectable before bacterial challenge. All organ weights are presented as a percentage of total body weight. The spleens were an average of 26% smaller in mutant compared to wild type mice (*p* = 0.002) (Figure 1(a)). To determine whether the observed difference in spleen size was due to the absence or reduction of any particular leukocyte subtype, flow cytometry was performed using a broad range of cell markers for common leukocyte subsets. No differences were detected between the leukocyte composition of naïve mutant spleens compared to naïve wild type spleens (Figure 1(b)). The spleen size differences were not due to changes in cell size, as cell size in mutant compared to wild type spleens was also not different based on the forward scatter mean fluorescent intensity of cells (see Supplemental Figure  1 in Supplementary Material available online at http://dx.doi.org/10.1155/2015/975789).

### 3.2. nBmp2NLS^tm^ Mice Responded Normally to Systemic Primary Bacterial Infection

**Figure 2:**
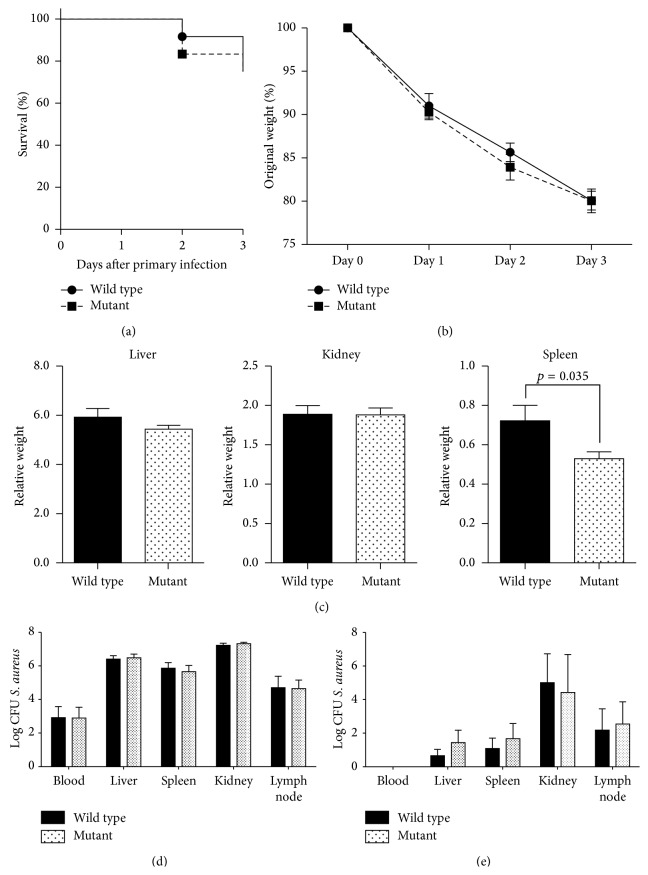
nBmp2NLS^tm^ mice responded normally to primary systemic infection with* S. aureus*. (a)–(d) Mice were infected by tail vein injection with 3 × 10^5^ CFU/g* S. aureus* and followed up for 3 days. (a) Percent survival (*n* = 12 per group). (b) Weight loss is plotted as average percent of original body weight ± SE (*n* = 12 per group). (c) Average liver, kidney, and spleen weights as a percentage of total body weight ± SE on day 3 after primary infection (*n* = 12 per group). (d) Various tissues were cultured on day 3 after primary infection to measure bacterial load, and results shown are average CFU/mL ± SE for blood samples and average CFU/g ± SE for the other tissues (*n* = 12 per group). (e) Mice were infected by tail vein injection with 1 × 10^4^ CFU/g* S. aureus*, a dose that caused no mortality. Tissues were cultured on day 8 after primary infection to measure bacterial load, and results shown are average CFU/mL ± SE for blood samples and average CFU/g ± SE for liver, spleen, kidney, and lymph node samples (*n* = 4 for wild type and *n* = 3 for mutant mice).

Wild type and nBmp2NLS^tm^ micewere infected systemically with 3 × 10^5^ CFU/g* S. aureus* on day 0. Mice were weighed daily and were sacrificed on day 3 to measure bacterial load in various organs. Both wild type and nBmp2NLS^tm^ mice showed ~25% mortality by day 3 (Figure 2(a)). The surviving wild type and mutant mice lost ~20% of their body weight by day 3 (Figure 2(b)). Three days after infection, the weights of liver and kidney were similar in mutant and wild type mice. As seen in naïve mice, the spleen weight of nBmp2NLS^tm^ mice was lower than wild type mice after primary infection (Figure 2(c)). Cultures from blood, liver, spleen, kidney, and lymph nodes on day 3 after primary infection showed no difference in the bacterial load between wild type and nBmp2NLS^tm^ mice (Figure 2(d)).

Primary* S. aureus* infections were repeated at a lower dose of bacteria (1 × 10^4^ CFU/g). This infectious dose caused no mortality in either group of mice, and no differences in bacterial load were observed upon sacrifice at day 8 (Figure 2(e)). Furthermore, no bacteria were cultured from blood samples of either mutant or wild type mice at day 8 after infection. These data suggested that both wild type and nBmp2NLS^tm^ mice possessed equivalent primary immune responses to* S. aureus* at both high and low doses of bacteria.

### 3.3. Differences in Leukocyte Composition between nBmp2NLS^tm^ and Wild Type Mice Were Detectable in Spleen and Lymph Nodes after Primary Infection

**Figure 3:**
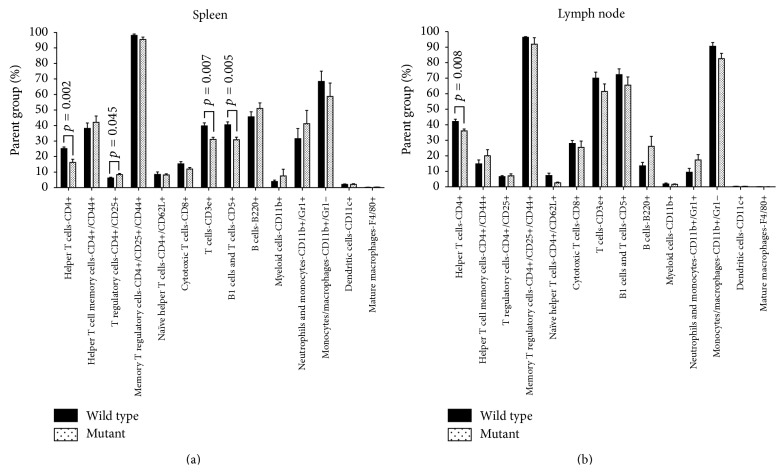
The relative abundance of certain cell types in the spleen and lymph nodes was altered after primary infection. Mice were infected by tail vein injection with 1 × 10^4^ CFU/g* S. aureus*, a dose that caused no mortality. (a) Spleen and (b) lymph nodes were harvested on day 8 after primary infection, and their leukocyte cell type composition was analyzed by flow cytometry. Values shown are average ± SE percent of parent group, as explained in the legend for Figure 1 (*n* = 5 per group).

Although nBmp2NLS^tm^ mice cleared bacteria after a primary infection as effectively as wild type mice, flow cytometric analysis revealed some small yet statistically significant differences in the leukocyte composition of nBmp2NLS^tm^ mouse spleens compared to wild type controls at 8 days after primary infection. Specifically, spleens of mutant mice exhibited a 35% decrease in helper T cells (CD4+), a 22% decrease in CD3e+ T cells, a 24% decrease in CD5+ B and T cells, and a 35% increase in T regulatory cells (CD4+/CD25+) (Figure 3(a)). A 15% decrease in the number of CD4+ T cells was seen in the lymph nodes of nBmp2NLS^tm^ mice compared to wild type controls (Figure 3(b)). These findings suggested that although there was no difference in the ability of mutant and wild type mice to clear a primary infection, there are slight differences in the prevalence of lymphocyte subsets in select lymphoid tissues.

### 3.4. nBmp2NLS^tm^ Mice Exhibited Defective Secondary Responses to* S. aureus*

**Figure 4:**
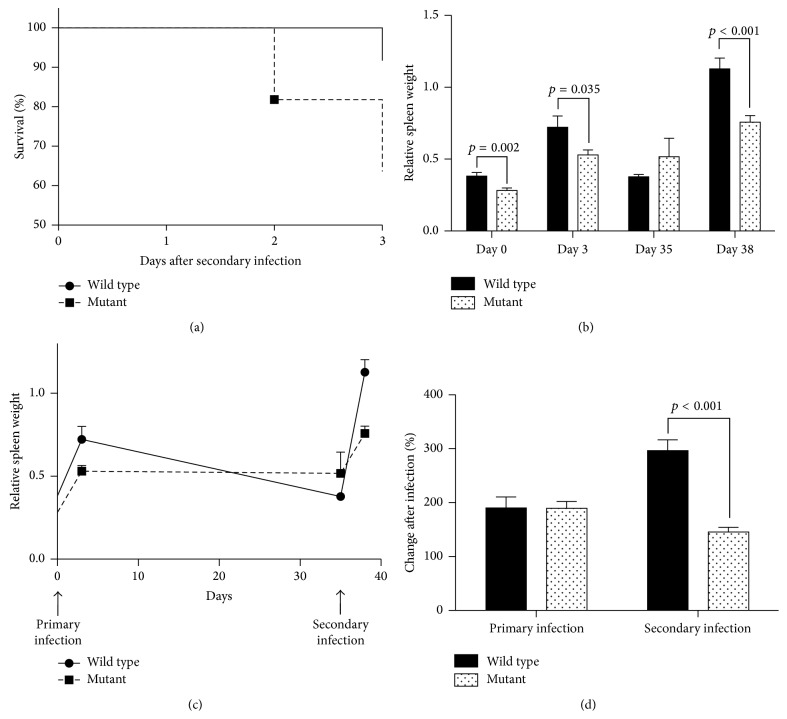
nBmp2NLS^tm^ mice showed increased mortality and impaired spleen enlargement after secondary infection. Mice were infected by tail vein injection with 1 × 10^4^ CFU/g* S. aureus *on day 0 and again with 3 × 10^5^ CFU/g* S. aureus* on day 35. (a) Percent survival for 3 days after secondary infection (*n* = 12 for wild type and *n* = 11 for mutant mice). (b) Spleen weight as a percentage of total body weight on day 38 (3 days after secondary infection) compared to day 0 (naïve mice), day 3 (3 days after primary infection), and day 35 (immediately before secondary infection) (*n* = range from 7 to 20 per group). (c) Same data as in (b), but presented in line graph format to emphasize the more dramatic changes in wild type compared to mutant spleens. (d) Spleen weight changes following primary and secondary infections, showing relative spleen weight on days 3 and 38 as a percentage of relative spleen weight on days 0 and 35, respectively.

To examine the response of wild type and mutant mice to a secondary infection challenge, mice were infected systemically with a priming dose of* S. aureus* at 1 × 10^4^ CFU/g on day 0 and then infected again with a higher dose of 3 × 10^5^ CFU/g on day 35. Spleen weight, bacterial load, and leukocyte composition of lymph nodes, spleen, and thymus were measured on day 38. All mice survived the primary infection; however, nBmp2NLS^tm^ mice exhibited greater mortality following secondary infection when compared to wild type (Figure 4(a)).

On day 38, 3 days after secondary infection, spleens were again significantly smaller in nBmp2NLS^tm^ mice compared to wild type mice (as was seen in naïve mice and following primary infection) (Figure 4(b)). Dramatic differences between wild type and mutant animals were seen in the magnitude of spleen size expansion and contraction over the course of the experiment. Wild type spleens enlarged 1.9-fold by 3 days after the primary infection, returned to original weight by day 35, and then enlarged 3-fold by 3 days after the secondary infection. Although spleens from mutant animals similarly enlarged 1.9-fold by 3 days after the primary infection, they failed to decrease in weight by day 35 as was seen in wild type animals (Figures 4(b) and 4(c)). Following secondary infection, mutant spleens increased only 1.5-fold (Figures 4(b) and 4(c)). This difference is illustrated clearly by comparing postinfection as a percentage of preinfection spleen size in wild type and mutant mice—mutant mice show impaired spleen expansion only after secondary infection (Figure 4(d)).

### 3.5. Bacterial Load in the Blood of nBmp2NLS^tm^ Mice Was Increased following Secondary Infection

**Figure 5:**
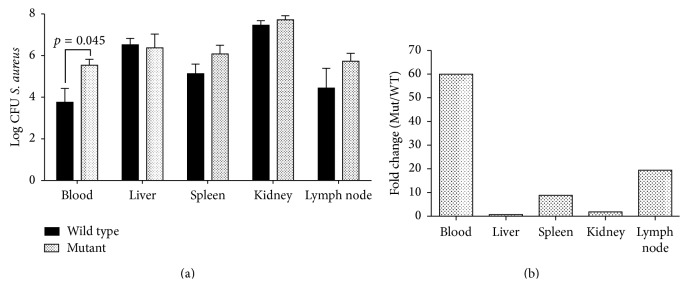
Clearance of bacteria from the bloodstream was impaired in nBmp2NLS^tm^ mice. Tissues were harvested and cultured on day 38 (3 days after secondary infection) to measure bacterial load. (a) Average CFU/mL ± SE for blood samples and average CFU/g ± SE for liver, spleen, kidney, and lymph node samples, shown on a log scale (*n* = 6 per group). (b) The same data as in (a), but shown as the fold difference of nBmp2NLS^tm^ compared to wild type, to emphasize the 60-fold increase in blood bacterial load.

Bacterial load in blood from nBmp2NLS^tm^ mice was increased 60-fold compared to wild type mice by three days after secondary infection (Figures 5(a) and 5(b)). This dramatic increase suggested that mutant mice were less effective than wild type mice at clearing bacteria from the blood stream following secondary infection. Bacterial loads in the liver, spleen, kidneys, and lymph nodes of nBmp2NLS^tm^ mice were not significantly different than wild type following secondary infection (Figure 5(a)).

### 3.6. Helper and Cytotoxic T Cell Populations Were Reduced in nBmp2NLS^tm^ Lymph Nodes after Secondary Infection

**Figure 6:**
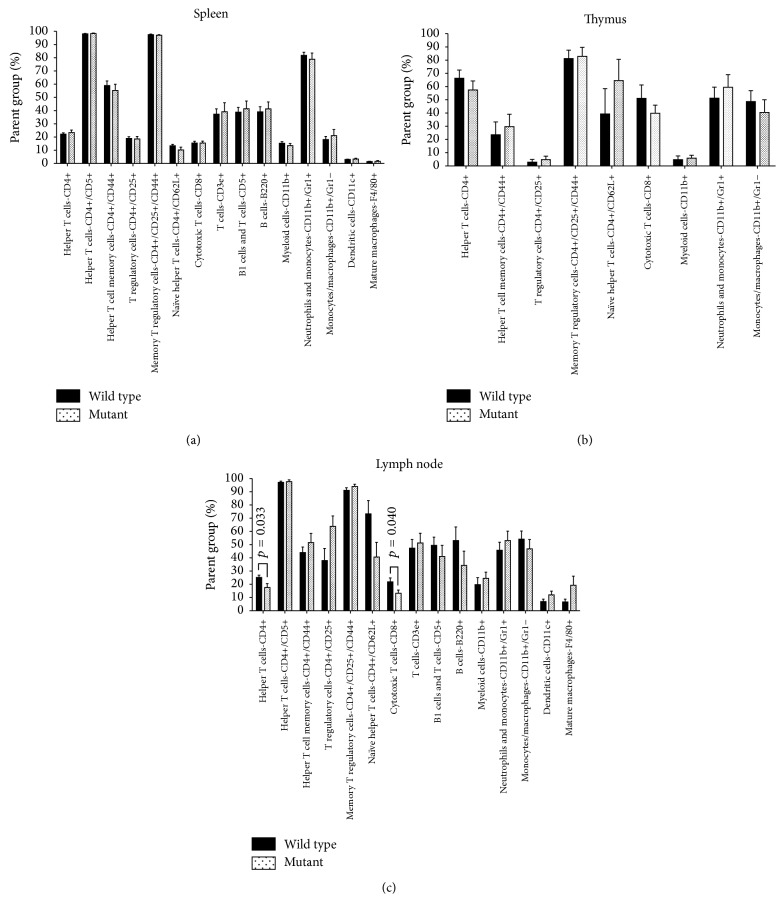
Lymph nodes in nBmp2NLS^tm^ mice showed changes in relative leukocyte composition compared to wild type after secondary infection. Leukocyte cell type composition of spleen (a), thymus (b), and lymph nodes (c) from wild type and nBmp2NLS^tm^ mice was measured on day 38 (3 days after secondary infection) using flow cytometry. Values shown are average ± SE percent of parent group, as explained in the legend of Figure 1 (*n* = 5 per group).

Based on the dramatic differences seen following secondary infection in mouse mortality and bacteremia, we analyzed leukocyte populations in spleen, thymus, and lymph nodes from surviving mice on day 38, three days after secondary infection. No significant differences in the relative numbers of any leukocyte populations in the spleen were observed (Figure 6(a)), suggesting that the ineffective clearance of bacteria from the blood stream is not due to a lack or depletion of any major leukocyte subset in the spleen. Additionally, no difference in leukocyte subset composition was observed in the thymus (Figure 6(b)). Lymph nodes, however, did have some small differences in the relative numbers of some lymphocyte subsets: helper T cells (CD4+) were reduced by 30% and cytotoxic T cells (CD8+) were reduced by 39% in lymph nodes from nBmp2NLS^tm^ mice compared to wild type (Figure 6(c)).

### 3.7. Calcium Flux Was Not Significantly Different between nBmp2NLS^tm^ and Mutant Lymphocytes

**Figure 7:**
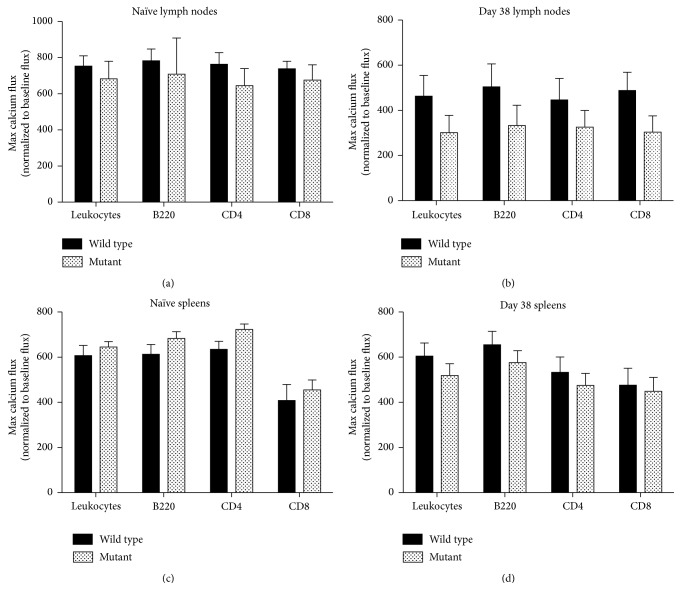
Calcium flux was not significantly reduced in nBmp2NLS^tm^ compared to wild type lymph node or spleen leukocytes. Spleens (a and b) and lymph nodes (c and d) were harvested from wild type and nBmp2NLS^tm^ mice on day 0 (naïve mice, a and c) and on day 38 (3 days after secondary infection, b and d). Calcium flux was measured using ionomycin stimulation of Fluo4-loaded cells in total leukocytes and in B220+ (B cells), CD4+ (helper T cells), and CD8+ (cytotoxic T cells) subgroups. Results shown are average ± SE (*n* = 8 per group, except for day 38 mutants, *n* = 9).

Based on the hypothesis that calcium signaling may be compromised in nBmp2NLS^tm^ mice, we next assayed for defects in calcium mobilization within the cells of wild type and mutant mice. The fluorescent calcium indicator Fluo4 was used in conjunction with ionomycin stimulation to measure calcium flux in lymphocytes from naïve and infected mice (day 38). No differences in calcium flux were detected in lymph node or splenic B cells, CD4+ T cells, or CD8+ T cells from naïve mice or following secondary infection (Figures 7(a)–7(d)). Lymph node lymphocytes harvested after secondary infection showed a trend toward decreased calcium flux in nBmp2NLS^tm^ compared to wild type mice, but the differences were not statistically significant (Figure 7(d)).

### 3.8. Serum Antibody Titers Were Not Different in nBmp2NLS^tm^ Compared to Wild Type Mice

**Figure 8:**
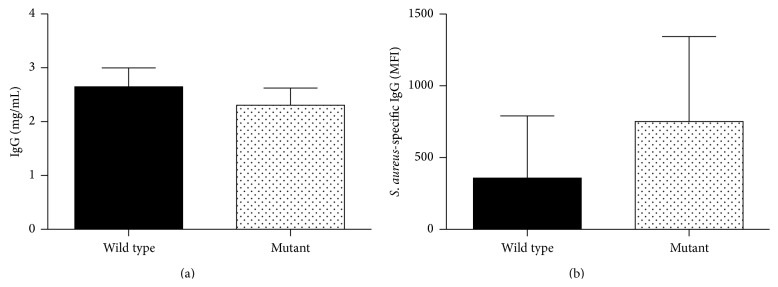
Serum antibody titers were not different between wild type and nBmp2NLS^tm^ mice following secondary infection. (a) Serum was harvested from wild type and nBmp2NLS^tm^ mice on day 38 (3 days after secondary infection), and IgG antibody concentrations were measured by ELISA. Results shown represent the average ± SE (*n* = 9 for wild type, *n* = 11 for mutant). (b) Antigen-specific antibody levels were determined using flow cytometry to measure the binding of serum IgG to intact* S. aureus* as described in Section 2.5. No statistically significant differences were seen in the levels of antigen-specific IgG from the serum of mutant compared to wild type mice. Results shown represent the average mean fluorescence intensity ± SE.

Previous research has shown that genetic ablation of PPAR*γ* (a member of the nuclear hormone receptor superfamily) resulted in global defects in the* in vitro* production of select antibody isotypes [8]. To determine if a generalized decrease in antibody response and accompanying decrease in opsonization potential might account for the reduced survival and clearance of bacteria after secondary infection in nBmp2NLS^tm^ mice, we next determined levels of serum antibodies. No significant antibody differences between wild type and mutant mice were detected in naïve mice (data not shown). Following secondary infections, levels of total IgG, IgM, and IgA antibody were determined. No significant differences were seen in the total IgG, IgM, or IgA antibody levels, suggesting no defect in production of these antibody isotypes (Figure 8(a) and data not shown). We next investigated the possibility that although total antibody levels were unchanged, antigen-specific antibody levels may be affected in mutant mice. As IgG is the predominant isotype of antibody seen in the serum following secondary infection, we assayed for relative levels of antigen-specific IgG in the serum of wild type and mutant mice following secondary infection. Flow cytometric analysis showed no difference in the levels of* S. aureus*-specific IgG following secondary infection in nBmp2NLS^tm^ compared to wild type mice (Figure 8(b)).

### 3.9. Spleens of Mutant Mice Were Structurally Similar to Wild Type but Had Fewer Hemosiderin-Laden Macrophages

**Figure 9:**
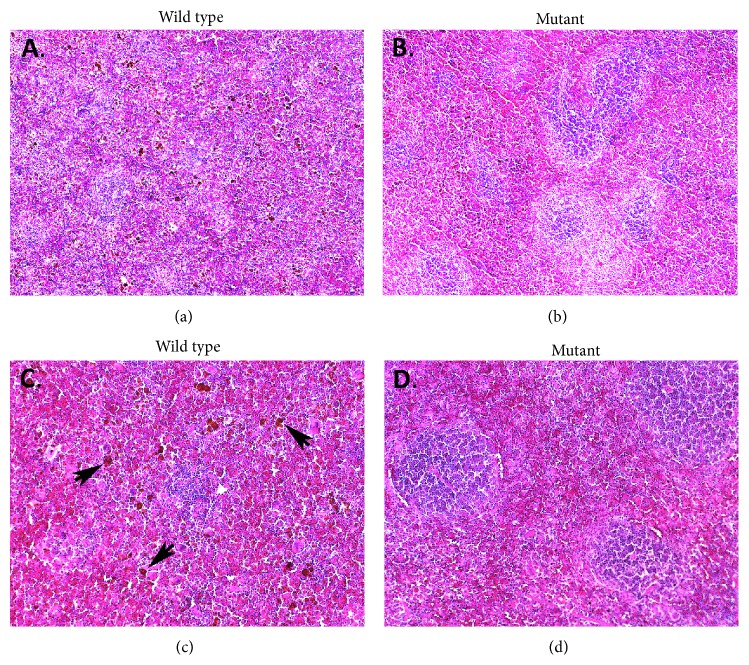
Mutant spleens were structurally similar to wild type but had fewer hemosiderin-laden macrophages. Spleens were harvested from wild type (a, c) and mutant (b, d) mice on day 38 (3 days after secondary infection). They were fixed, embedded in paraffin, stained with H&E, and examined using brightfield microscopy. Images were captured through 5x (a and b) and 10x (c and d) objectives. Hemosiderin-laden macrophages appear as large, rust-colored cells (some are marked with arrows) in these representative sections.

We examined spleens histologically three days after secondary infection, using H&E staining. The structure and arrangement of red pulp and white pulp regions did not appear different between wild type and mutant mice, nor did overall cellularity. We did, however, observe fewer hemosiderin-laden macrophages in mutant compared to wild type spleens (Figure 9).

## 4. Discussion

BMP2 is a well-studied growth factor involved in patterning and cellular fate in various organs [9–19]. Our recent description of a novel nuclear variant of BMP2, nBMP2, raised the question of whether functions previously attributed to secreted BMP2 might instead be mediated by nBMP2, since both proteins can be translated from the same mRNA transcript and therefore coexpressed by the same cell [1]. To address this question, we generated a novel mouse line (nBmp2NLS^tm^) with targeted disruption of nBMP2 but normal secretion of BMP2 [2].

The focus of this study was to determine if nBmp2NLS^tm^ mutant mice have an impaired immune response to systemic bacterial infection. Such an impairment was originally hypothesized on the basis that nBmp2NLS^tm^ mice show Ca^2+^-related skeletal muscle dysfunction and therefore might also show Ca^2+^-related immune dysfunction [2, 5, 9, 20–22]. In addition, BMP proteins are known to play a role in thymus morphogenesis and T cell activation and differentiation, indicating that the potential for nBMP2 expression exists in immune system cells [22, 23]. We found that mutation of nBMP2 does, in fact, disrupt the secondary immune response in nBmp2NLS^tm^ mice, resulting in high levels of bacteremia in the nBmp2NLS^tm^ mice and dramatically decreased survival in response to secondary* S. aureus* infection. Thus, the recently described nuclear variant of BMP2 is essential for normal secondary immune responses.

In an effort to elucidate the potential mechanism of this impaired immune response we performed extensive flow cytometric analysis of leukocyte subsets, lymphocyte calcium analysis, and characterization of immune organ weight changes. In our analysis of uninfected animals we observed decreased spleen weight in mutant compared to wild type animals. Detailed analysis also demonstrated that mutant mice showed additional small differences in leukocyte populations in spleen and lymph nodes after primary systemic* S. aureus* infection. Mutant and wild type mice responded similarly to the primary infection in terms of mortality, weight loss, spleen enlargement, and bacterial load.

Importantly, wild type mice developed immunity through the priming dose that protected them against a higher secondary dose. This was evident by decreased mortality following a high dose of* S. aureus* if it was preceded by a priming dose (the high dose infection alone was lethal to 25% of the wild type mice, but mortality dropped to 8% when the high dose was preceded by the priming dose). Mutant mice, in contrast, showed 25% mortality from the high dose infection alone but mortality increased to 36% when it was preceded by the priming dose. These results strongly suggest that although relatively minor differences were seen in the distribution of lymphocyte subsets in wild type and mutant mice before infection or after primary infection, mutant mice do differ in immunologically significant ways, which result in a dramatic increase in mortality following secondary bacterial exposure. Additionally, data showing a 60-fold increase in the bacterial count in blood of mutant compared to wild type mice further demonstrate that nBmp2 is essential for clearance of* S. aureus* following secondary infection.

Enlargement of the spleen is a characteristic feature of the systemic inflammatory response to many different pathogens [24–29]. Although the mechanism of the inability of nBmp2NLS^tm^ mice to mount effective protection against secondary bacterial challenge remains enigmatic, the small but significant differences in some T lymphocyte populations after primary infection may provide a clue. We found no size difference between cells in mutant compared to wild type spleens, either before or after infection, suggesting that the lack of splenic enlargement seen in mutant mice after secondary infection could result from a lack of effective trafficking of immune cells to the spleen or failure to proliferate once arrived.

Other possibilities for the immune defect seen in nBmp2NLS^tm^ mice include a lack of effective phagocytosis/killing of bacteria by macrophages or neutrophils. Such a defect could be a result of failure to efficiently opsonize bacteria or the inability to effectively kill phagocytized cells once engulfed. We saw no differences in IgM, IgG, and IgA levels, but changes in the number of hemosiderin-laden macrophages suggest that nBmp2NLS^tm^ mice do exhibit changes in some phagocytic cell functions. Flow cytometric analysis demonstrated that macrophages numbers were not reduced in mutant mouse spleens. Future studies are needed to measure the phagocytic activity of macrophages in the spleens of nBmp2NLS^tm^ mice, to determine whether there might be a functional deficit in macrophage phagocytic activity or trafficking defects [30].

## 5. Conclusions

In summary, we report that nBmp2NLS^tm^ mice have an immune system defect that is manifest by decreased ability to clear bacteria from the bloodstream and increased mortality after a secondary systemic bacterial infection. This is the first demonstration of an immune related function for nBMP2. Although the mechanism of this action remains to be elucidated, here we demonstrate that it is not dependent on ineffective calcium mobilization in lymphocytes, inability to mount an antigen-specific immune response, or lack of a major leukocyte subset.

## Supplementary Material

Supplemental Figure 1. Splenic cell sizes in wild type and mutant mice are identical. A) Forward scatter and side scatter plot for a representative uninfected (naïve) wild type spleen. B) Forward scatter histogram of the same representative naïve wild type spleen as in A, showing the two populations of gated cells (FSC A-, smaller cells; FSC-A+, larger cells). C) Forward scatter and side scatter plot of a representative naïve nBmp2NLStm mutant spleen. D) Forward scatter histogram of the same representative naïve nBmp2NLStm mutant spleen as in C, showing the two populations of gated cells (FSC A-, smaller cells; FSC-A+, larger cells). E) Mean fluorescent intensity for the two gates (FSC A- and A+) comparing the sizes of naïve wild type and mutant spleen cells (n=10). F) Mean fluorescent intensity for the two gates (FSC A- and A+) comparing the sizes of wild type and mutant spleen cells 8 days after primary infection (n=5). G) Mean fluorescent intensity for the two gates (FSC A- and A+) comparing the sizes of wild type and mutant spleen cells 3 days after secondary infection (and 38 days after primary infection) (n=10). Values shown are average ± SE.

## Figures and Tables

**Table 1:** Antibodies used for leukocyte identification.

	Marker	Stain	Antibody	Clone	eBioscience reference number
Group 1	CD11b	FITC	Anti-mouse CD11b FITC	M1/70	11-0112-82
	CD8a	PE	Anti-mouse CD8a PE	53-6.7	12-0081-82
	CD4	APC	Anti-mouse CD4 eFluor 660	GK1.5	50-0041-82
	Gr-1	PE-Cy7	Anti-mouse Ly-6g (Gr-1) PE-Cy7	RB6-8C5	25-5931-82

Group 2	B220	FITC	Anti-human/Mouse CD45R (B220) FITC	RA3-6B2	11-0452-82
	CD8a	PE	Anti-mouse CD8a PE	53-6.7	12-0081-82
	CD4	APC	Anti-mouse CD4 eFluor 660	GK1.5	50-0041-82
	Gr-1	PE-Cy7	Anti-mouse Ly-6g (Gr-1) PE-Cy7	RB6-8C5	25-5931-82

Group 3	CD11c	FITC	Anti-mouse CD11c FITC	N418	11-0114-82
	CD5	PE	Anti-mouse CD5 PE	53-7.3	12-0051-82
	CD4	APC	Anti-mouse CD4 eFluor 660	GK1.5	50-0041-82
	Gr-1	PE-Cy7	Anti-mouse Ly-6g (Gr-1) PE-Cy7	RB6-8C5	25-5931-82

Group 4	F4/80	FITC	Anti-mouse F4/80 FITC	BM8	11-4801-82
	CD3e	PE	Anti-mouse CD3e PE	145-2C11	12-0031-82
	CD4	APC	Anti-mouse CD4 eFluor 660	GK1.5	50-0041-82
	Gr-1	PE-Cy7	Anti-mouse Ly-6g (Gr-1) PE-Cy7	RB6-8C5	25-5931-82

Group 5	CD4	FITC	Anti-mouse CD4 FITC	GK1.5	11-0041-82
	CD44	PE	Anti-human/Mouse CD44 PE	IM7	12-0441-82
	CD25	APC	Anti-mouse CD25 APC	PC61.5	17-0251-82
	Gr-1	PE-Cy7	Anti-mouse Ly-6g (Gr-1) PE-Cy7	RB6-8C5	25-5931-82
	CD62L	APC-Cy7	Anti-mouse CD62L APC-eFluor 780	MEL-14	47-0621-82

## References

[1] Felin J. E., Mayo J. L., Loos T. J. (2010). Nuclear variants of bone morphogenetic proteins. *BMC Cell Biology*.

[2] Bridgewater L. C., Mayo J. L., Evanson B. G. (2013). A novel bone morphogenetic protein 2 mutant mouse, nBmp2NLS^tm^, displays impaired intracellular Ca^2+^ handling in skeletal muscle. *BioMed Research International*.

[3] Odermatt A., Barton K., Khanna V. K. (2000). The mutation of Pro^789^ to Leu reduces the activity of the fast-twitch skeletal muscle sarco(endo)plasmic reticulum Ca^2+^ ATPase (SERCA1) and is associated with Brody disease. *Human Genetics*.

[4] Odermatt A., Taschner P. E. M., Khanna V. K. (1996). Mutations in the gene-encoding SERCA1, the fast-twitch skeletal muscle sarcoplasmic reticulum Ca^2+^ ATPase, are associated with Brody disease. *Nature Genetics*.

[5] Feske S. (2007). Calcium signalling in lymphocyte activation and disease. *Nature Reviews Immunology*.

[6] Lewis R. S. (2001). Calcium signaling mechanisms in T lymphocytes. *Annual Review of Immunology*.

[7] Vig M., Kinet J.-P. (2009). Calcium signaling in immune cells. *Nature Immunology*.

[8] Ramon S., Bancos S., Thatcher T. H. (2012). Peroxisome proliferator-activated receptor *γ* b cell-specific-deficient mice have an impaired antibody response. *The Journal of Immunology*.

[9] Hager-Theodorides A. L., Outram S. V., Shah D. K. (2002). Bone morphogenetic protein 2/4 signaling regulates early thymocyte differentiation. *Journal of Immunology*.

[10] Chen D., Zhao M., Harris S. E., Mi Z. (2004). Signal transduction and biological functions of bone morphogenetic proteins. *Frontiers in Bioscience*.

[11] Goldstein A. M., Brewer K. C., Doyle A. M., Nagy N., Roberts D. J. (2005). BMP signaling is necessary for neural crest cell migration and ganglion formation in the enteric nervous system. *Mechanisms of Development*.

[12] Hogan B. L. M. (1996). Bone morphogenetic proteins: multifunctional regulators of vertebrate development. *Genes and Development*.

[13] Kanzler B., Foreman R. K., Labosky P. A., Mallo M. (2000). BMP signaling is essential for development of skeletogenic and neurogenic cranial neural crest. *Development*.

[14] Maatouk D. M., Choi K.-S., Bouldin C. M., Harfe B. D. (2009). In the limb AER Bmp2 and Bmp4 are required for dorsal-ventral patterning and interdigital cell death but not limb outgrowth. *Developmental Biology*.

[15] Mishina Y. (2003). Function of bone morphogenetic protein signaling during mouse development. *Frontiers in Bioscience*.

[16] Reversade B., Kuroda H., Lee H., Mays A., De Robertis E. M. (2005). Depletion of Bmp2, Bmp4, Bmp7 and Spemann organizer signals induces massive brain formation in Xenopus embryos. *Development*.

[17] Schlange T., Arnold H.-H., Brand T. (2002). BMP2 is a positive regulator of Nodal signaling during left-right axis formation in the chicken embryo. *Development*.

[18] Wozney J. M. (1992). The bone morphogenetic protein family and osteogenesis. *Molecular Reproduction and Development*.

[19] Zhang H., Bradley A. (1996). Mice deficient for BMP2 are nonviable and have defects in amnion/chorion and cardiac development. *Development*.

[20] Graf D., Nethisinghe S., Palmer D. B., Fisher A. G., Merkenschlager M. (2002). The developmentally regulated expression of Twisted gastrulation reveals a role for bone morphogenetic proteins in the control of T cell development. *The Journal of Experimental Medicine*.

[21] Lu L., Ma J., Wang X. (2010). Synergistic effect of TGF-*β* superfamily members on the induction of Foxp3^+^ Treg. *European Journal of Immunology*.

[22] Yoshioka Y., Ono M., Osaki M., Konishi I., Sakaguchi S. (2012). Differential effects of inhibition of bone morphogenic protein (BMP) signalling on T-cell activation and differentiation. *European Journal of Immunology*.

[23] Hager-Theodorides A. L., Ross S. E., Sahni H., Mishina Y., Furmanski A. L., Crompton T. (2014). Direct BMP2/4 signaling through BMP receptor IA regulates fetal thymocyte progenitor homeostasis and differentiation to CD4+CD8+ double-positive cell. *Cell Cycle*.

[24] Koc A., Bargen I., Suwandi A. (2014). Systemic and mucosal immune reactivity upon mycobacterium avium ssp. paratuberculosis infection in mice. *PLoS ONE*.

[25] Singh V. K., Srivastava M., Dasgupta A., Singh M. P., Srivastava R., Srivastava B. S. (2014). Increased virulence of *Mycobacterium tuberculosis* H37Rv overexpressing LipY in a murine model. *Tuberculosis*.

[26] Logan L. K., Jacobs N. M., McAuley J. B., Weinstein R. A., Anderson E. J. (2011). A multicenter retrospective study of childhood brucellosis in Chicago, Illinois from 1986 to 2008. *International Journal of Infectious Diseases*.

[27] Silva T. M. A., Costa E. A., Paixo T. A., Tsolis R. M., Santos R. L. (2011). Laboratory animal models for brucellosis research. *Journal of Biomedicine and Biotechnology*.

[28] Tseng Y.-T., Sheng W.-H., Lin B.-H. (2011). Causes, clinical symptoms, and outcomes of infectious diseases associated with hemophagocytic lymphohistiocytosis in Taiwanese adults. *Journal of Microbiology, Immunology and Infection*.

[29] Tarantino G., Scalera A., Finelli C. (2013). Liver-spleen axis: intersection between immunity, infections and metabolism. *World Journal of Gastroenterology*.

[30] Masuda T., Satodate R., Tsuruga K., Kasai T. (1993). Quantitative assessment of a change of hemosiderin deposition with age in splenic compartments of rats. *Tohoku Journal of Experimental Medicine*.

