# Supplementary material for: Targeted Mutation of Nuclear Bone Morphogenetic Protein 2 Impairs Secondary Immune Response in a Mouse Model
Source: Biomed Res Int. 2015 Aug 23;2015:975789. doi: 10.1155/2015/975789 (PMC4603606; doi:10.1155/2015/975789)
Supplement: Supplementary file 1 — Supplemental Figure 1. Splenic cell sizes in wild type and mutant mice are identical. A) Forward scatter and side scatter plot for a representative uninfected (naïve) wild type spleen. B) Forward scatter histogram of the same representative naïve wild type spleen as in A, showing the two populations of gated cells (FSC A-, smaller cells; FSC-A+, larger cells). C) Forward scatter and side scatter plot of a representative naïve nBmp2NLStm mutant spleen. D) Forward scatter histogram of the same representative naïve nBmp2NLStm mutant spleen as in C, showing the two populations of gated cells (FSC A-, smaller cells; FSC-A+, larger cells). E) Mean fluorescent intensity for the two gates (FSC A- and A+) comparing the sizes of naïve wild type and mutant spleen cells (n=10). F) Mean fluorescent intensity for the two gates (FSC A- and A+) comparing the sizes of wild type and mutant spleen cells 8 days after primary infection (n=5). G) Mean fluorescent intensity for the two gates (FSC A- and A+) comparing the sizes of wild type and mutant spleen cells 3 days after secondary infection (and 38 days after primary infection) (n=10). Values shown are average ± SE. [file 975789.f1.pdf]

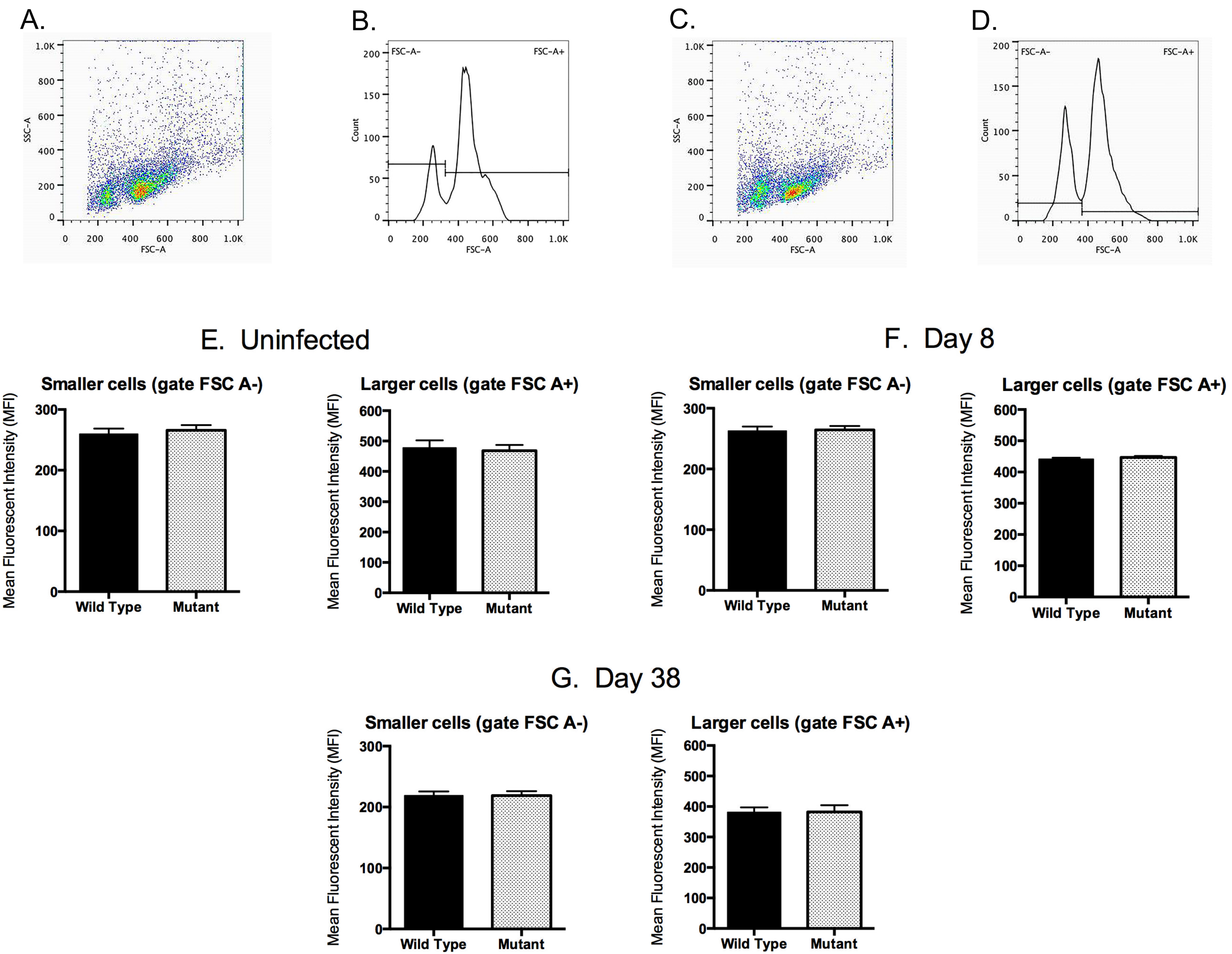

A.
SSC-A
FSC-A
B.
FSC-A-
FSC-A+
Count
FSC-A
C.
SSC-A
FSC-A
D.
FSC-A-
FSC-A+
Count
FSC-A
E. Uninfected
Smaller cells (gate FSC A-)
Larger cells (gate FSC A+)
Mean Fluorescent Intensity (MFI)
Wild Type
Mutant
F. Day 8
Smaller cells (gate FSC A-)
Larger cells (gate FSC A+)
Mean Fluorescent Intensity (MFI)
Wild Type
Mutant
G. Day 38
Smaller cells (gate FSC A-)
Larger cells (gate FSC A+)
Mean Fluorescent Intensity (MFI)
Wild Type
Mutant

Supplemental Figure 1. Splenic cell sizes in wild type and mutant mice are identical. A) Forward scatter and side scatter plot for a representative uninfected (naïve) wild type spleen. B) Forward scatter histogram of the same representative naïve wild type spleen as in A, showing the two populations of gated cells (FSC A-, smaller cells; FSC-A+, larger cells). C) Forward scatter and side scatter plot of a representative naïve nBmp2NLS^{tm} mutant spleen. D) Forward scatter histogram of the same representative naïve nBmp2NLS^{tm} mutant spleen as in C, showing the two populations of gated cells (FSC A-, smaller cells; FSC-A+, larger cells). E) Mean fluorescent intensity for the two gates (FSC A- and A+) comparing the sizes of naïve wild type and mutant spleen cells (n=10). F) Mean fluorescent intensity for the two gates (FSC A- and A+) comparing the sizes of wild type and mutant spleen cells 8 days after primary infection (n=5). G) Mean fluorescent intensity for the two gates (FSC A- and A+) comparing the sizes of wild type and mutant spleen cells 3 days after secondary infection (and 38 days after primary infection) (n=10). Values shown are average ± SE.
